# Clinical Retrospective Study on Superior Capsular Reconstruction Using Autologous Fascia Lata Combined With LARS Ligament for Massive Irreparable Rotator Cuff Tears

**DOI:** 10.1111/os.70307

**Published:** 2026-04-13

**Authors:** Fangbing Zhu, Weibin Du, Hongfeng Ruan, Meng Ge, Gang Qu, Yanghua Tang, Zhengcong Ye, Shigui Yan

**Affiliations:** ^1^ Department of Orthopedic Surgery, the Second Affiliated Hospital Zhejiang University School of Medicine Hangzhou Zhejiang China; ^2^ Research Institute of Orthopedics The Jiangnan Hospital Affiliated to Zhejiang Chinese Medical University Hangzhou Zhejiang China; ^3^ Institute of Orthopaedics and Traumatology The First Affiliated Hospital of Zhejiang Chinese Medical University (Zhejiang Provincial Hospital of Traditional Chinese Medicine) Hangzhou Zhejiang China

**Keywords:** arthroscopy, artificial ligament, fascia lata, massive irreparable rotator cuff tear, superior capsule reconstruction

## Abstract

**Objective:**

Massive irreparable rotator cuff tears cause severe shoulder dysfunction. Superior capsular reconstruction (SCR) is effective, but optimal graft material is controversial; combined autologous fascia lata and LARS ligament needs validation.

**Methods:**

We retrospectively analyzed 30 patients who underwent superior capsular reconstruction using autologous fascia lata combined with LARS ligament for massive irreparable rotator cuff tears between November 2023 and May 2025. We evaluated American Shoulder and Elbow Surgeons (ASES) scores, University of California, Los Angeles (UCLA) scores, Constant‐Murley (CMS) scores, visual analogue scale (VAS) scores, shoulder range of motion, and acromiohumeral distance (AHD). Paired *t*‐tests or Wilcoxon signed‐rank tests were used to compare preoperative and postoperative outcomes, and *p* < 0.05 was considered statistically significant. And analyzed the complications after the operation.

**Results:**

All 30 patients were followed up for a mean duration of months (12.35 ± 2.13). Seven cases involved isolated irreparable supraspinatus tendon tears, 19 cases had combined supraspinatus and infraspinatus tendon tears, and 4 cases included subscapularis tendon tears. In 3 cases, the long head of the biceps tendon was fixed to replace the anterior aspect of the supraspinatus tendon; in the remaining cases, the long head of the biceps tendon was transected. No glenoid fossa deformity was observed. At final follow‐up, all functional scores improved significantly (all *p* < 0.05): ASES score increased from 31.04 ± 4.26 to 82.12 ± 3.23, UCLA score from 11.83 ± 4.15 to 30.44 ± 1.83, and CMS score from 32.44 ± 6.72 to 83.42 ± 4.75. The VAS score decreased from 5.84 ± 0.87 to 1.22 ± 0.42 (*p* < 0.05). Active range of motion in shoulder forward flexion, abduction, external rotation, and internal rotation improved significantly (all *p* < 0.05). Postoperative AHD significantly increased from 4.25 ± 0.41 mm to 10.15 ± 0.16 mm (*p* < 0.05). No complications such as wound infection, nerve injury, anchor loosening, lateral thigh pain, hematoma, or numbness occurred.

**Conclusion:**

For patients with massive irreparable rotator cuff tears, superior capsular reconstruction using autologous fascia lata combined with LARS ligament effectively alleviates shoulder pain, enhances glenohumeral stability, and improves shoulder function.

## Graphical abstract

For patients with dysfunctional irreparable rotator cuff tears, superior capsular reconstruction using autologous fascia lata combined with LARS artificial ligament effectively restores shoulder function, significantly improves range of motion, and demonstrates a favorable safety profile.

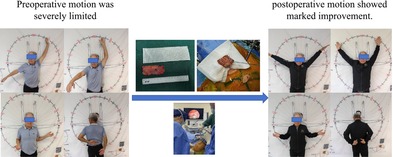

## 1 Introduction

Rotator cuff tears represent a common shoulder disorder in orthopedics and sports medicine, with an incidence that increases significantly with age. Among individuals over 60 years old, the prevalence can exceed 30% [1, 2]. Massive irreparable rotator cuff tears (MIRCT) present a significant challenge in shoulder surgery due to their extensive tear size, poor tendon quality, and muscle atrophy. Massive rotator cuff tears account for 10%–40% of all rotator cuff tears, while irreparable tears constitute 6.5%–22.4% of massive tears. The failure rate after conventional repair surgery reaches 94%, with a re‐tear rate as high as 80% [3, 4]. Patients suffer from prolonged shoulder pain and restricted movement, leading to a severe decline in quality of life. Due to loss of superior shoulder joint stability, irreparable large rotator cuff tears cause severe functional impairment [5, 6]. Statistics indicate that after nonsurgical treatment, only 56% of patients experience pain relief, 94% still report weakness in forward flexion, and 56% develop periarthritic muscle atrophy, failing to meet patients' quality‐of‐life expectations [7, 8, 9].

For irreparable massive rotator cuff tears, surgical options are diverse, including arthroscopic debridement, long head of biceps tendon (LHBT) tenotomy or tenodesis, greater tuberosity tuberoplasty, reverse shoulder arthroplasty, partial rotator cuff repair, tendon transfer, augmentation with biologic or synthetic patches, and subacromial balloon spacer [10, 11, 12, 13, 14]. However, each approach has significant limitations. Superior capsule reconstruction (SCR), first proposed in 2013 by Mihata et al. [15], reconstructs the superior capsule of the glenohumeral joint to restrict humeral head upward migration, thereby improving shoulder mobility. It has become a significant option in shoulder‐preserving surgery. However, the core challenge of this procedure lies in graft selection: while autologous fascia lata offers excellent biocompatibility and promotes tissue healing, it suffers from insufficient initial mechanical strength and the risk of long‐term “graft creep,” making it difficult to maintain shoulder stability over time. Conversely, synthetic ligaments alone can provide immediate and robust mechanical support but raise questions about biocompatibility, often triggering foreign body reactions or chronic synovitis, thereby compromising long‐term outcomes [16, 17, 18].

Thus, identifying an ideal SCR graft combining sufficient mechanical strength with high biocompatibility has become a critical clinical priority. Ding et al. [19] modified autologous fascia lata grafts by inserting an artificial ligament between two folded layers of autologous fascia lata, creating a ‘sandwich’ graft. This design theoretically mimics a “reinforced concrete” structure to achieve superior clinical outcomes. LARS artificial ligaments, composed of polyethylene terephthalate, feature a highly porous structure conducive to tissue ingrowth, potentially providing an ideal mechanical and biological platform for composite grafts [20, 21, 22]. However, clinical reports on the efficacy of this composite technique remain scarce, and its safety, effectiveness, and long‐term stability in treating massive irreparable rotator cuff tears have not been fully validated.

The purpose of this retrospective study was to: (i) evaluate the clinical efficacy of SCR using autologous fascia lata combined with LARS ligament in patients with MIRCT; (ii) assess functional and imaging outcomes including shoulder function scores, range of motion, pain relief, acromiohumeral distance restoration, and graft integration; and (iii) provide clinicians with evidence‐based guidance for graft selection in SCR procedures.

## 2 Materials and Methods

### 2.1 Study Population

A retrospective analysis was conducted on 30 patients (19 males, 11 females) treated with SCR using autologous fascia lata combined with LARS ligament for MIRCT between November 2023 and May 2025. Injury location: right shoulder (20 cases), left shoulder (10 cases). Indications for surgery: 28 cases of chronic degenerative tears and 2 cases of traumatic tears. The research was conducted in accordance with the principles of the Declaration of Helsinki. All patients provided written informed consent. The IEC No. 2024090.

### 2.2 Inclusion and Exclusion Criteria

Patients were included if they met the following criteria: (1) Preoperative MRI confirmed massive rotator cuff tear (involving ≥ 2 tendons or a single tendon with tear length ≥ 5 cm) meeting irreparable criteria (tendon retraction > 2 cm, severe muscle fatty infiltration on preoperative MRI, particularly Goutallier grade 3–4 in the infraspinatus muscle) [23, 24]; (2) No severe glenohumeral osteoarthritis on preoperative radiographs; (3) Arthroscopy confirmed irreparability of supraspinatus and infraspinatus tendons, but reparability of the subscapularis tendon; (4) Preoperative physical examination revealed intact deltoid muscle function; (5) Relatively young patients with high functional demands and pseudo‐paralysis are unsuitable for reverse shoulder arthroplasty; (6) First‐time shoulder surgery; (7) Postoperative follow‐up ≥ 1 year with complete clinical and imaging data.

#### 2.2.1 Exclusion Criteria

(1) Shoulder instability or infection; (2) Severe glenohumeral subluxation uncorrectable by arm traction; (3) Cervical nerve root or axillary nerve palsy; (4) High risk of anchor screw loosening due to severe osteoporosis; (5) Surgical contraindications such as severe cardiovascular disease or coagulation disorders; (6) Poor compliance, including failure to adhere to postoperative rehabilitation protocols or loss to follow‐up.

### 2.3 Surgical Approach

(1) Under general anesthesia with brachial plexus nerve block, the patient was positioned in lateral decubitus with adequate exposure of the affected shoulder. Perform routine disinfection and draping of the surgical field. Disinfect and drape the ipsilateral thigh. A 5 mm skin incision was made 2 cm posterior‐inferior to the lateral angle of the acromion and 1 cm medial to it. A blunt‐tipped needle was advanced toward the coracoid process to enter the glenohumeral joint.

A shoulder arthroscope was inserted to sequentially examine intra‐articular structures, assessing: (1) Tear or degeneration of the long head of the biceps tendon; (2) integrity of the subscapularis muscle and feasibility of repair; (3) retraction, elasticity, and degree of fatty infiltration of the supraspinatus and infraspinatus tendon tears; (4) extent of degeneration and wear of the humeral head and glenoid cartilage. Synovectomy and rotator interval release were performed as needed.

After withdrawing the arthroscope, a blunt‐tipped needle was advanced through the original incision toward the anterolateral aspect of the acromion to enter the subacromial space. The arthroscope was reinserted to examine the supraspinatus and infraspinatus tendons, assess subscapularis tear severity, evaluate acromion morphology and impingement severity, and assess the degree of osteophytic formation on the greater tuberosity of the humerus.

Subacromial bursectomy was performed to visualize the subacromial space. For Type II and Type III acromions, acromioplasty was performed, particularly on the anterior and lateral acromion borders and the acromioclavicular joint. Reverse acromioplasty is indicated for osteophytes on the greater tubercle of the humerus. Release the supraspinatus tendon, subscapularis tendon, and subscapularis muscle. Greater tuberosity decompression was performed if osteophytes were present.

After debriding the footprint area, the subscapularis tendon was repaired first if torn (using a single‐row or double‐row technique). Partial rotator cuff repair was then performed to restore anterior–posterior force couple balance, which is key to SCR technique success. Subsequently, the Neviaser portal was established. Based on the size of the superior capsular defect, one or two 3.5‐mm threaded anchors (Johnson & Johnson) were implanted at the glenoid superior rim. The medial‐anterior diameter, lateral‐anterior diameter, medial‐lateral diameters of the defect area, and the distance between the two anchors were measured to determine the appropriate graft dimensions.

(2) A longitudinal incision was made in the upper‐middle third of the lateral thigh on the affected side. The skin and subcutaneous tissue were dissected layer by layer to expose the fascia lata. An appropriate size of autologous fascia lata (adjustments are made on an individual basis based on factors such as the size of the patient's rotator cuff defect) was harvested. After irrigation, the wound was closed in layers.

After trimming the autologous fascia lata, two layers of LARS artificial ligament (LARS, France; model LARS‐SRK) were placed in the center. These layers were woven into a “sandwich”‐like composite graft according to the defect size (the final thickness was approximately 8 mm).

The lateral portal was expanded, and the composite graft was delivered into the subacromial space using anterior and posterior traction sutures. Graft tension was adjusted to ensure no excessive laxity or tension when the shoulder joint was in the neutral position. Secure it to the glenoid fossa and the humeral greater tuberosity bed. The graft was secured to the glenoid using the previously placed anchors. A double‐row suture bridge technique was used to anchor the graft to the humeral greater tuberosity. After arthroscopically confirming reliable graft fixation and absence of impingement during shoulder movement, irrigate the joint cavity and close the surgical approach. When intraoperative arthroscopic visualization is poor, 0.05 mL epinephrine (Shanghai Hefeng Pharmaceutical Co. Ltd., 1 mg/mL) is added to 3 L of irrigation fluid to maintain continuous irrigation.

(3) Postoperatively, one vial of ropivacaine (AstraZeneca, 75 mg/10 mL), half a vial of compound betamethasone (Shanghai Schering‐Plow Pharmaceuticals Co. Ltd., 1 mL: 5 mg), and one vial of sodium hyaluronate (Shanghai Haohai Biotech Co. Ltd., 2 mL: 20 mg) injected into the glenohumeral joint and subacromial space. Portal sites were infiltrated with liposomal bupivacaine (Jiangsu Hengrui Medicine Co. Ltd., 133 mg/10 mL) for postoperative anesthesia. No drainage tube was used. Postoperatively, routine ice application was performed. Antibiotics were used within 24 h. Celecoxib was taken orally for pain relief.

### 2.4 Postoperative Management

The affected shoulder was immobilized in a shoulder brace at 30° abduction and neutral position for 8 weeks. On the second postoperative day, patients were instructed to begin finger grip exercises and elbow range‐of‐motion exercises. After 2 weeks, pendulum exercises and supine passive forward flexion exercises were introduced, while avoiding active shoulder movement. At 4 weeks postoperatively, standing passive forward flexion and passive external rotation training were gradually initiated. Active forward flexion exercises were added after 6 weeks, with range‐of‐motion limited to 90°. Wall‐climbing exercises were introduced at 8 weeks, followed by passive internal rotation and extension exercises at 10–12 weeks. Resistance training was gradually increased after 12 weeks. Patients resumed daily activities at 6 months, while avoiding heavy labor and strenuous exercise.

### 2.5 Outcome Assessments

The following outcomes were evaluated preoperatively and at final follow‐up: (1) Imaging evaluation: MRI examination to assess rotator cuff healing (presence of re‐tears, graft integration status). (2) Functional scores: American Shoulder and Elbow Surgeons (ASES) score (maximum 100 points), University of California, Los Angeles Shoulder Score (UCLA) score (maximum 35 points), American Shoulder and Elbow Surgeons (ASES) score (maximum 100 points), University of California, Los Angeles Shoulder (UCLA) score (maximum 35 points), and Constant‐Murley Shoulder (CMS) score (maximum 100 points). (3) Range of motion measurements: Record active shoulder joint range of motion, including forward flexion, abduction, external rotation, and internal rotation. (4) Acromiohumeral distance (AHD) measured on anteroposterior shoulder X‐ray. (5) Surgical complications. The scoring was performed by a senior orthopedic graduate student who was not involved in the study.

### 2.6 Statistical Analysis

Statistical analysis was performed using SPSS version 24.0 software (IBM Corp., Armonk, NY, USA). Normality of continuous variables was assessed using the Shapiro–Wilk test. Normally distributed data are presented as mean ± standard deviation, and non‐normally distributed data are presented as Median. Paired *t*‐tests were used to compare preoperative and postoperative outcomes for normally distributed data, the Wilcoxon rank‐sum tests were used for non‐normally distributed data. *p* < 0.05 was considered statistically significant.

## 3 Results

### 3.1 Patient Demographics and Surgical Characteristics

All 30 patients were followed up for a mean duration of 12.35 ± 2.13. No complications occurred, including wound infection, nerve injury, anchor loosening, lateral thigh pain, hematoma, or numbness. Arthroscopic assessment revealed 18 Type II acromions and 12 Type III acromions. Seven cases involved isolated irreparable supraspinatus tendon tears, 19 cases had combined supraspinatus and infraspinatus tendon tears, and 4 cases included subscapularis tendon tears. In 3 cases, the long head of the biceps tendon was fixed to replace the anterior aspect of the supraspinatus tendon; the remaining cases underwent long head of the biceps tendon tenotomy. No glenoid deformity was observed.

### 3.2 Clinical Outcomes

At final follow‐up, all functional scores improved significantly compared with preoperative values (all *p* < 0.05, Table 1). The ASES score increased from 31.04 ± 4.26 to 82.12 ± 3.23, the UCLA score was from 11.83 ± 4.15 to 30.44 ± 1.83, and CMS score from 32.44 ± 6.72 to 83.42 ± 4.75. Conversely, the VAS score decreased from 5.84 ± 0.87 to 1.22 ± 0.42 (*p* < 0.05). In parallel, the active range of motion for shoulder forward flexion, abduction, external rotation, and internal rotation improved significantly (all *p* < 0.05). Forward flexion increased from 68.25° ± 15.90° to 150.13° ± 13.30°, abduction from 60.63° ± 13.38° to 136.45° ± 12.44°, external rotation from 21.25° ± 12.43° to 53.63° ± 6.33°, and internal rotation from 25.43° ± 3.76° to 58.68° ± 5.58°. AHD significantly increased from 4.25 ± 0.41 mm to 10.15 ± 0.16 mm (*p* < 0.05) (see Tables 1 and 2 for details).

**TABLE 1:** Comparison of shoulder joint function scores and AHD index between preoperative and final follow‐up in 30 patients (mean ± SD).

Time	ASES	UCLA	CMS	VAS	AHD(mm)
Pre‐Op	31.04 ± 4.26	11.83 ± 4.15	32.44 ± 6.72	5.84 ± 0.87	4.25 ± 0.41
Last‐follow‐up	82.12 ± 3.23	30.44 ± 1.83	83.42 ± 4.75	1.22 ± 0.42	10.15 ± 0.16
*T* value	52.34	24.93	34.05	26.19	73.38
*p* value	< 0.0001	< 0.0001	< 0.0001	< 0.0001	< 0.0001

**TABLE 2:** Comparison of active range of motion in the shoulder joint between preoperative and final follow‐up in 30 patients (mean ± SD).

Time	Forward flexion (°)	Abduction (°)	External rotation (°)	Internal rotation (°)
Pre‐Op	68.25 ± 15.90	60.63 ± 13.38	21.25 ± 12.43	25.43 ± 3.76
Last‐follow‐up	150.13 ± 13.30	136.45 ± 12.44	53.63 ± 6.33	58.68 ± 5.58
*T* value	21.64	22.73	12.71	27.08
*p* value	< 0.0001	< 0.0001	< 0.0001	< 0.0001

### 3.3 Radiographic Assessments

**FIGURE 1:**
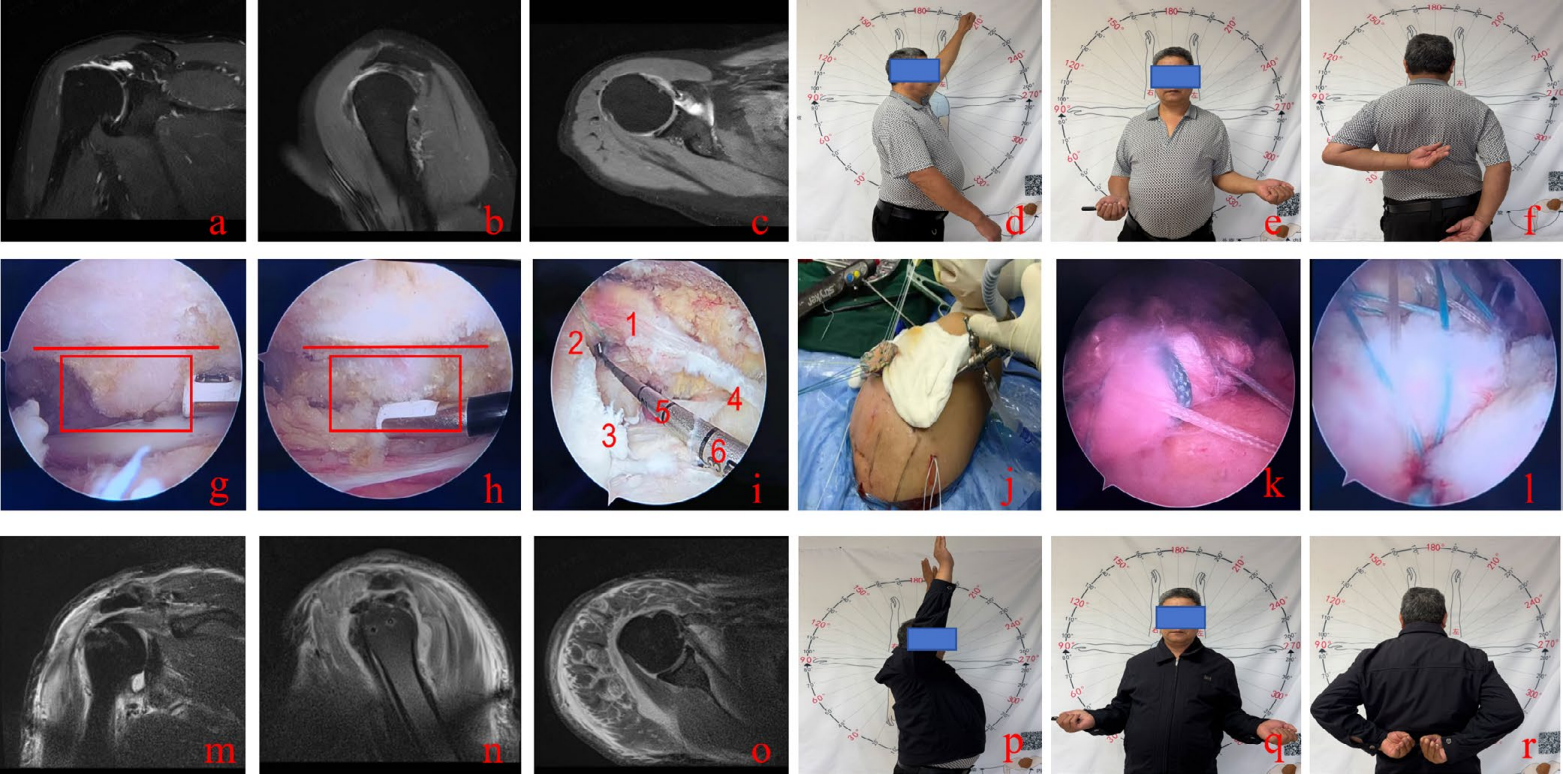
Patient, male, 68 years old, presenting with a massive irreparable rotator cuff tear. (a–c) Preoperative MRI demonstrating retraction of supraspinatus and subscapularis tears with upward displacement of the humeral head. (d–f) Preoperative shoulder joint range of motion during flexion, external rotation, and internal rotation. (g, h) Standardized acromioplasty under arthroscopic visualization during surgery. (i) 1–2: Anteroposterior diameter of supraspinatus defect medial aspect; 3–4: Anteroposterior diameter of supraspinatus defect lateral aspect; 2–5: Distance between two anchors in glenoid fossa and humeral footprint region; 2–6: Lateral diameter of supraspinatus defect area. (j–l) Intraoperative preparation of the “sandwich” patch, patch insertion into the supraspinatus defect, and rotator cuff repair. (m–o) Postoperative follow‐up MRI showing well‐repaired supraspinatus and subscapularis muscles, downward displacement of the humeral head, and good graft signal. (p–r) Postoperative follow‐up shoulder joint range of motion for flexion, external rotation, and internal rotation.

**FIGURE 2:**
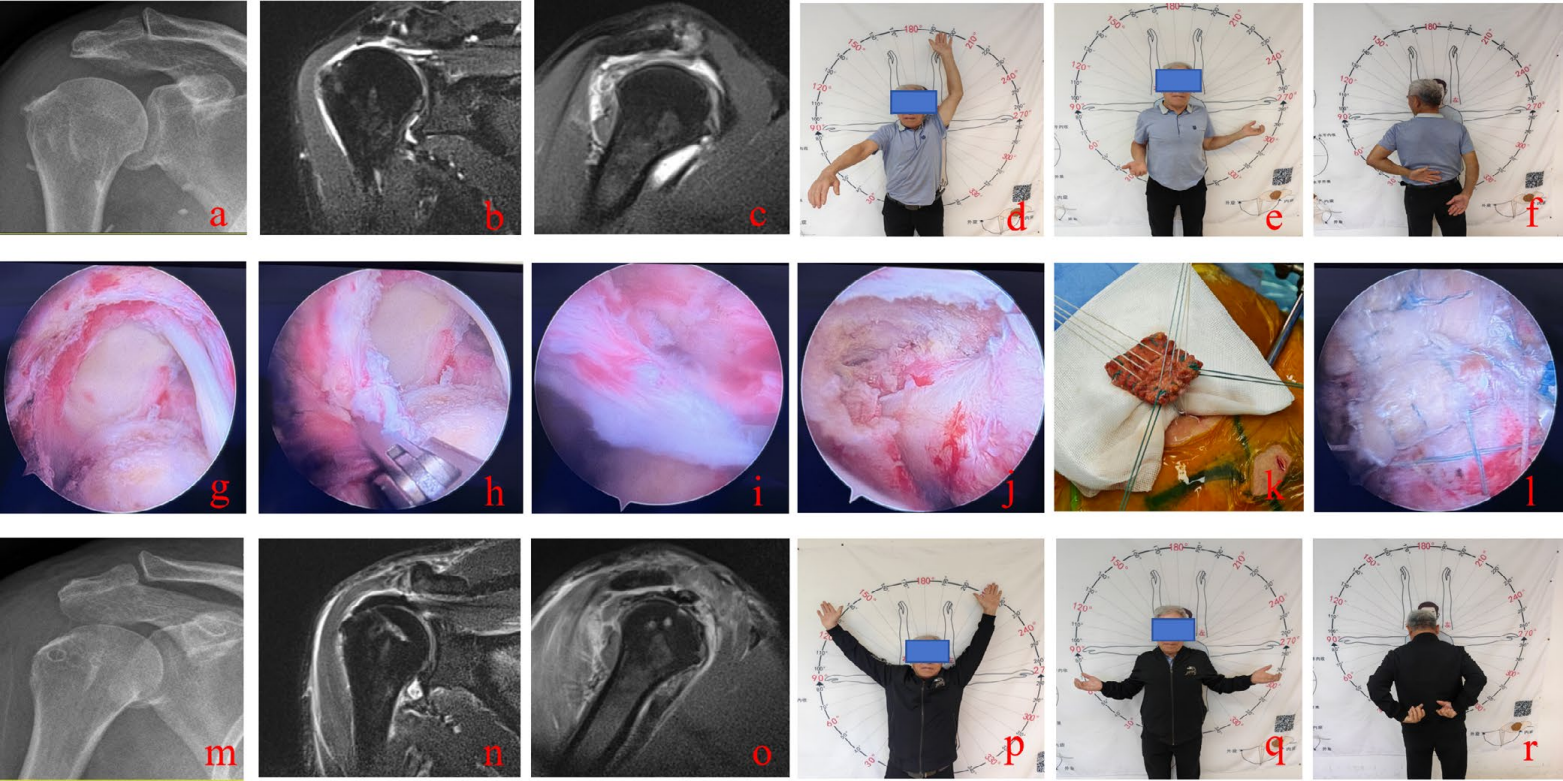
Patient, male, 57 years old, presenting with a massive irreparable rotator cuff tear. (a–c) Preoperative X‐ray and MRI showed a massive and irreparable rotator cuff injury, accompanied by upward displacement of the humeral head. (d–f) Preoperative shoulder joint range of motion during flexion, external rotation, and internal rotation. (g–j) Arthroscopy showed the conditions of the supraspinatus muscle, infraspinatus muscle, and subscapularis muscle. (k, l) Intraoperative preparation of the “sandwich” patch and rotator cuff repair. (m–o) The X‐ray and MRI results from the postoperative follow‐up examination showed that the rotator cuff repair was in good condition, the humeral head had shifted downward, and the transplanted tissue showed good signal. (p–r) Postoperative follow‐up shoulder joint range of motion for flexion, external rotation, and internal rotation.

At final follow‐up, MRI demonstrated excellent graft integration in 29 of 30 patients. One patient experienced a re‐tear (a partial tear of the supraspinatus muscle), but the patient did not report significant worsening of pain. The shoulder range of motion remained improved compared to preoperative levels, and no reoperation was performed. The typical cases are shown in Figures 1 and 2.

### 3.4 Complications

No significant complications such as wound infection, nerve injury, anchor loosening, lateral thigh pain, hematoma, or numbness were observed in the follow‐up patients. This may be attributed to the small sample size or insufficient follow‐up duration. However, it is noteworthy that these surgical complications have not occurred to date, coupled with the observed significant functional and structural improvements, which collectively confirm the favorable safety profile and clinical efficacy of this procedure.

## 4 Discussion

MIRCT presents a significant clinical challenge due to poor tissue quality, muscle atrophy, and high re‐tear rates following conventional repair. SCR has emerged as a promising joint‐preserving alternative, but optimal graft selection remains controversial: autologous fascia lata offers excellent biocompatibility and promotes tissue healing but lacks adequate initial mechanical strength and is susceptible to graft creep, while synthetic ligaments provide robust immediate mechanical support but raise concerns about long‐term biocompatibility and tissue integration. This study investigated a composite graft approach combining autologous fascia lata with LARS ligament to synergize the advantages of both materials while minimizing their respective limitations. Our results demonstrated significant improvements in functional outcomes, substantial pain relief, marked improvement in range of motion, and restoration of glenohumeral biomechanics, with only one partial re‐tear and no major complications. These findings suggest that the composite graft technique balances immediate mechanical stability with long‐term biological integration, representing a promising treatment option for this challenging patient population.

### 4.1 Composite Graft Design: Synergizing Mechanical Support With Biological Integration

The rationale for composite graft design stems from the complementary properties of its constituent materials. However, the long‐term survival rate of LARS ligament is lower than that of autologous or allogeneic tendons, particularly in young and highly active patients, where the risk of long‐term re‐tears may be higher [25, 26]. In this study, the autologous broad fascia combined with LARS ligament was used to repair the huge irreparable rotator cuff injury. Autologous broad fascia provides a biological repair foundation, promoting tendon‐bone healing, and the combination of both can form the advantages of “biological repair” and “mechanical stability”. Postoperative AHD increased from preoperative (4.25 ± 0.41) mm to (10.15 ± 0.16) mm, restoring to the normal physiological range (7–14 mm). consistent with the reported improvement trend in AHD following traditional SCR procedures [27, 28]. However, the increase in AHD was more pronounced in this study, likely attributed to the superior immediate mechanical support provided by the composite graft.

From a biological integration perspective, the incorporation of autologous fascia lata addressed the insufficient biocompatibility of pure artificial ligaments. Clinical follow‐up revealed only one case of partial supraspinatus muscle re‐tear among 30 patients, with no significant clinical symptoms. In terms of functional improvement, the postoperative ASES, UCLA, and CMS scores showed significant increases compared to the preoperative levels, while the VAS score decreased significantly. Notably, among the 3 patients in this study who underwent long head of biceps tendon fixation to replace the anterior supraspinatus tendon, no “Popeye Deformity” (anterior‐superior depression of the humeral head) developed postoperatively. This suggests that the modified technique may further optimize anterior‐superior shoulder stability and reduce abnormal postoperative biomechanical changes.

### 4.2 Superior Functional Recovery and Pain Relief With Composite Grafts

For massive irreparable rotator cuff tears, commonly used clinical treatment options each have limitations: Reverse shoulder arthroplasty rapidly improves pain but involves significant surgical trauma and exhibits higher prosthesis loosening rates in younger patients (< 65 years old) [29, 30]. Tendon transfer procedures (e.g., latissimus dorsi transfer) require sacrificing healthy tendons, may cause scapulothoracic joint dysfunction postoperatively, and demand highly specialized surgical expertise [31, 32]. While autologous fascia lata SCR offers excellent biocompatibility, graft creep rates can reach 15%–20% at 1 year postoperatively, predisposing to recurrent shoulder instability [3, 33].

In contrast, the composite graft technique employed in this study presents the following unique advantages: ① no healthy tendon sacrifice is required, eliminating donor site complications, ② incorporation of the LARS ligament reduces graft creep risk, with no graft loosening observed at the final follow‐up, and ③ minimal surgical trauma (intraoperative blood loss of approximately 50–100 mL) and low postoperative complication rate, lower than that of reverse shoulder arthroplasty. Furthermore, the postoperative rehabilitation period in this study was comparable to that of SCR alone (6 months to resume daily activities), but functional recovery was faster. Passive shoulder range of motion improved by 40%–50% at 4 weeks postoperatively compared to preoperative levels. This may be attributed to the enhanced early stability of the composite graft, allowing patients to initiate rehabilitation training sooner.

### 4.3 Restoration of Glenohumeral Biomechanics: AHD And Graft Healing

In this study, the postoperative acromiohumeral distance (AHD) significantly increased from 4.25 ± 0.41 mm to 10.15 ± 0.16 mm. This improvement in a key radiographic parameter directly confirms that this surgical technique effectively restores the superior physical barrier and sealing function of the shoulder joint, thereby reversing the tendency for superior humeral head migration caused by massive irreparable tears. The restoration of AHD is a core biomechanical indicator of surgical success. It contributes to reestablishing normal force couples across the glenohumeral joint, providing the structural foundation for postoperative pain relief and functional improvement [27, 34]. The fundamental concept of combining autologous fascia lata with the LARS ligament in this technique is as follows: the autologous fascia lata provides a matrix for biological healing, potentially facilitating host cell ingrowth and tissue remodeling to achieve long‐term biological integration. Conversely, the LARS ligament provides immediate and intermediate‐term stable structural support. Its high‐strength properties enable it to withstand the tensile forces generated during shoulder motion, thereby protecting the biological healing process.

### 4.4 Surgical Technique Refinement and Critical Technical Points

Systematic Arthroscopic Diagnosis and Pre‐Reconstruction Preparation: The procedure commences with a comprehensive intra‐articular and subacromial diagnostic examination. A thorough subacromial bursectomy and, when indicated, acromioplasty (for Type II/III acromions) or reverse acromioplasty (for greater tuberosity osteophytes) are performed to create an impingement‐free space for graft placement. This preparatory step is fundamental for preventing postoperative mechanical impingement and ensuring long‐term graft survival.

Graft Preparation and Composite Construct Fabrication: Harvesting an autologous fascia lata graft of adequate size and quality forms the biological foundation for healing. A key technical refinement lies in meticulously weaving the LARS artificial ligament within the fascia lata to create a “sandwich‐like” composite structure. This design facilitates a biomechanical and biological synergy: the LARS ligament provides immediate, reliable structural tension to resist superior humeral migration, while the enveloping autologous fascia lata serves as a scaffold for host cell ingrowth, vascularization, and ultimate biological integration.

Graft Implantation and Tensioning: This phase is critical for biomechanical restoration. First, glenoid‐sided fixation must be secure and anatomically precise (at the superior glenoid rim) to reconstruct the anatomical insertion of the superior capsule, which is the basis for restoring the concavity‐compression effect. Second, humeral‐sided fixation using a double‐row suture bridge technique provides broad footprint coverage and robust compression. The most technically demanding step is tension adjustment: the graft must be fixed under appropriate tension with the shoulder in a neutral or slightly abducted position. This ensures the graft provides stable support in adduction without excessively restricting motion or risking fixation failure in abduction.

Phased and Individualized Postoperative Rehabilitation Protocol: Surgical success is equally dependent on a stringent postoperative regimen. The adopted protocol employs a phased rehabilitation strategy: Immediate strict protection with an abduction brace for 8 weeks, permitting elbow, wrist, and hand exercises to reduce edema. Pendulum exercises are initiated at 2 weeks, followed by the gradual introduction of passive forward flexion and external rotation training between 4 and 6 weeks. The core principle during this phase is to protect the graft from tensile forces generated by active muscle contraction. Active‐assisted and strengthening exercises are deferred until after 8–12 weeks, allowing for initial graft incorporation. This graduated protocol is designed to balance the early need to prevent joint stiffness with the imperative to protect the graft from premature mechanical loading, representing a vital component for optimizing clinical outcomes.

### 4.5 Comprehensive Comparative Analysis With Existing Mainstream Surgical Techniques

1. Compared to Isolated Autologous Fascia Lata Reconstruction: Isolated fascia lata grafting relies on the inherent healing and remodeling capacity of biological tissue. Its mechanical strength during the early maturation phase is relatively limited, carrying a risk of stretching or failure under tensile load, which often necessitates a longer and more stringent period of postoperative immobilization [35]. Our technique addresses this by incorporating the LARS ligament as a core mechanical scaffold, providing reliable immediate and intermediate structural support. This “biomechanical composite” design aims to protect the biological healing process and theoretically permits earlier initiation of safe, controlled rehabilitation, potentially reducing the clinical failure rate associated with premature mechanical loading.
2. Compared to Tendon Transfer (e.g., Latissimus Dorsi or Lower Trapezius Transfer): Tendon transfer aims to restore partial shoulder function through dynamic reconstruction. Its success is highly dependent on preserved innervation of the transferred muscle, precise tensioning, and intensive patient reeducation. This procedure alters the native shoulder kinematics, demands high technical expertise, and results in variable functional outcomes [36]. Our technique represents a combined static‐dynamic reconstruction. It restores the glenohumeral mechanical environment by reconstructing the passive stabilizing structure of the superior capsule (static stability), while preserving the function of the patient's remaining rotator cuff muscles, particularly the subscapularis (dynamic stability).
3. Compared to Arthroscopic Balloon Spacer Implantation: Absorbable subacromial balloon spacers [37, 38] (e.g., InSpace) function by occupying space to create an interpositional effect, passively depressing the humeral head to partially restore glenohumeral alignment. Its advantages include minimal invasiveness and relative procedural simplicity. However, its mechanism is one of pure physical interposition and spacing. It does not achieve biological integration or active capsular sealing. Its long‐term efficacy depends on the quality of the surrounding fibrous scar tissue formed after device resorption, and it carries risks such as device migration, rupture, or foreign‐body reaction. In contrast, our technique aims for active biological integration and mechanical reconstruction.
4. Compared to Reverse Total Shoulder Arthroplasty (RSA): RSA is a well‐established and effective procedure for end‐stage shoulder pathology. However, it is fundamentally a non‐anatomical joint replacement surgery. It achieves prosthetic stability and deltoid‐mediated function by altering the biomechanical center of rotation, at the cost of sacrificing normal joint anatomy [39]. Our technique is a joint‐preserving reconstructive procedure, with the core objective of repairing and reconstructing the patient's own anatomy.

### 4.6 Safety Profile and Complication Management

Based on this study's experience, the following key points should be noted when clinically applying autologous fascia lata composite LARS ligament‐superior capsule reconstruction: (1) Preoperative MRI must accurately assess rotator cuff tear extent, muscle‐fat infiltration (Goutallier grading), and subscapularis tendon function to strictly control surgical indications; (2) Intraoperatively, ensure moderate graft tension. Excessive laxity may cause postoperative instability, while excessive tightness may restrict shoulder joint mobility. Maintain mild tension in the neutral position. (3) When harvesting autologous fascia lata, control incision length (< 5 cm) to avoid injury to the lateral femoral cutaneous nerve. Apply compression bandaging postoperatively to prevent hematoma formation. (4) Postoperative rehabilitation should be progressive. Avoid active shoulder abduction and external rotation for 8 weeks to prevent graft loosening. Gradually increase resistance training intensity after 12 weeks.

### 4.7 Study Limitations and Future Directions

This study has several limitations that warrant consideration. (1) The retrospective design introduces selection bias, as all included patients had intact deltoid function and reparable subscapularis tendons, limiting the generalizability of our findings to the entire MIRCT population. (2) The small sample size (30 cases) and short follow‐up duration (mean 12.35 months) limit our ability to detect rare complications and to assess long‐term graft durability. Further observation of long‐term outcomes (e.g., graft stability and re‐tear rates at 3–5 years) is essential to determine whether the excellent early results are sustained over time. (3) The absence of control groups (e.g., LARS ligament SCR alone, autologous fascia lata SCR) precludes direct comparison of graft efficacy and makes it impossible to definitively attribute the favorable outcomes to the composite graft design rather than to patient selection, surgical technique, or rehabilitation protocols. (4) Long‐term imaging follow‐up (e.g., 2‐year postoperative MRI assessment of graft degradation) was absent, precluding clarification of the composite graft's long‐term biological changes.

Given these limitations, future research may explore the following directions: ① conduct multicenter, prospective randomized controlled trials to compare the efficacy differences between composite grafts and traditional grafts, ② expand sample size and extend follow‐up duration to evaluate the long‐term safety and efficacy of composite grafts, and ③ further optimize composite graft weaving techniques through biomechanical experiments (e.g., adjusting LARS ligament layers or autologous fascia thickness) to achieve superior mechanical properties and biological integration.

## 5 Conclusion

In summary, this retrospective study demonstrates that SCR using autologous fascia lata combined with LARS ligament effectively alleviates shoulder pain, enhances glenohumeral stability, and improves shoulder function in patients with MIRCT.

## Author Contributions

**Fangbing Zhu:** conceptualization, data curation, funding acquisition, methodology, writing – original draft. **Weibin Du:** conceptualization, data curation, formal analysis, writing – original draft. **Hongfeng Ruan:** conceptualization, formal analysis, data curation, writing – original draft. **Meng Ge:** methodology, formal analysis, validation. **Gang Qu:** methodology, formal analysis, validation. **Yanghua Tang:** methodology, formal analysis, validation. **Zhengcong Ye:** methodology, validation, formal analysis. **Shigui Yan:** supervision, methodology, writing – review and editing, project administration.

## Funding

This research was supported by Zhejiang Province Medical and Health Science and Technology Project (No. 2025KY191, 2025HY0801, 2023KY235). Hangzhou Bio‐medicine and Health Industry Development Support Science and Technology Project (No. 2023WJC243, 2023WJC249, 2023WJC234). Hangzhou Science and Technology Planning Project (No. 20241029Y120).

## Ethics Statement

This study was approved by the Ethics Committee of Jiangnan Hospital affiliated to Zhejiang Chinese Medical University (Approval NO. 2024090). The study was conducted in accordance with the principles of the Declaration of Helsinki.

## Consent

Written informed consent was obtained from all participants prior to their enrollment in this study.

## Conflicts of Interest

The authors declare no conflicts of interest.

## Data Availability

The data that support the findings of this study are available on request from the corresponding author. The data are not publicly available due to privacy or ethical restrictions.
